# Effects of Culture Aeration and the C:N Ratio on Propagule Production by Submerged Cultivation of *Clonostachys rosea* and Its Antifungal Metabolite Profiling

**DOI:** 10.1002/mbo3.70162

**Published:** 2025-11-17

**Authors:** Gabriel Moura Mascarin, Márcia Regina Assalin, Nilce Naomi Kobori, Wagner Bettiol

**Affiliations:** ^1^ Embrapa Environment Jaguariúna Sao Paulo Brazil; ^2^ Consultant Researcher Jaguariúna Sao Paulo Brazil

**Keywords:** biofungicide, *Botrytis cinerea*, microgranular formulation, postharvest disease, secondary metabolites

## Abstract

*Clonostachys rosea* is a necrotrophic mycoparasite studied for biocontrol of plant pathogenic fungi, including *Botrytis cinerea*, the causal agent of gray mold that causes economic losses in several common fruits and vegetables. This study evaluated how the culture aeration, manipulated through the medium‐to‐flask volume ratio, affects the submerged production of conidia and microsclerotia, key propagules for disease control. A low medium‐to‐flask ratio (1:5), which enhances aeration, significantly increased propagule yields. A high C:N ratio (50:1) favored submerged conidia production under elevated aeration, while microsclerotia formed only with low C:N (10:1) and boosted under high aeration. These propagules, along with cell‐free culture filtrates, were formulated into water‐dispersible microgranules and tested for efficacy against gray mold on cherry tomatoes. All formulations reduced disease incidence. UPLC ESI–QTOF–MS analysis of the organic extract from the culture filtrate revealed sorbicillinoids as the major antifungal metabolites. Overall, this study highlights the role of aeration in optimizing *C. rosea* submerged cultivation and supports the potential of its propagules and metabolites for use in biocontrol strategies against postharvest disease induced by *B. cinerea*.

## Graphical abstract

Optimized submerged fermentation of *Clonostachys rosea* under high aeration enhances production of submerged conidia and microsclerotia. Formulated propagules and antifungal metabolites, including sorbicillinoids, effectively control tomato gray mold, offering a promising strategy for biocontrol product development.

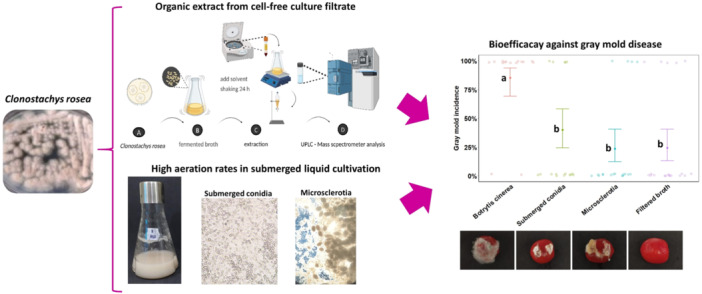

## 1 Introduction

The high demand for sustainable agriculture, with increasingly reduced chemical usage, has made biological control a high‐tech strategy in all agricultural systems, accompanied by an increased demand of farmers to explore and incorporate beneficial microbes in their agricultural practices (van Lenteren et al. 2018, 2025; Medeiros and Bettiol 2023). The bioinput market has grown by an average of 14.3% per year worldwide (Research and Markets 2024). The demand for biological control has increased due to the shift in the plant disease control paradigm in recent years. Previously, the goal was to completely eradicate pathogens by adopting chemical products, which resulted in the selection of pathogen isolates resistant to the main active ingredients. Additionally, it has led to the occurrence of outbreaks of diseases considered secondary, a reduction in beneficial microorganisms, damage to human and animal health, and environmental pollution, with the accumulation of residues in soil, water, and food (Aktar et al. 2009; Hahn 2014).

The necrotrophic mycoparasite fungus *Clonostachys rosea* (Ascomycota: Bionectriaceae) produces unicellular conidia on two types of conidiophores, namely, penicillate and verticillate, and is found in various types of soils and crop residues (Sun et al. 2020). This fungus also produces resistant propagules known as chlamydospores, which are more resistant to adverse environmental conditions than conidia (Sun et al. 2014, 2019). With a multitude of biocontrol mechanisms from lytic enzymes to secondary metabolites, *C. rosea* is considered a “jack of all trades” owing to its highly effective biocontrol activity against a wide range of plant pathogenic fungi, notably *Botrytis cinerea* (Cota et al. 2009; Karlsson et al. 2015; Jensen et al. 2022). Interestingly, this biocontrol agent also has insecticidal properties on some arthropod pests, such as the whitefly *Bemisia tabaci* (Mascarin et al. 2022), and has also been recognized for its nematicidal effects on plant parasitic nematodes, such as the root‐knot nematode *Meloidogyne incognita* (Cristóbal‐Alejo et al. 2021). In addition, this beneficial fungus can induce systemic resistance against pathogens and promote plant growth (Wang et al. 2019; Kamou et al. 2020; Jensen et al. 2022). Besides these roles, this fungus has endophytic ability, which is an important characteristic for controlling plant pathogens (Saraiva et al. 2015). Surprisingly, there are few commercial biopesticides based on *C. rosea* in the world market, and more specifically, this biocontrol agent is absent in the Brazilian market, the largest biopesticide market in the world (Jensen et al. 2022; Medeiros and Bettiol 2023).

For the viable commercialization of microorganisms, the choice of production method, nutritional composition, and abiotic conditions for optimal growth in the culture medium, alongside appropriate storage of the microbial biomass, and demonstration of the absence of risks both environmentally and in the handling of the microbial agent are together crucial for its success as a biopesticide (Elad and Stewart 2004). The mass production of fungi explored in pest biocontrol programs can occur through solid‐state, liquid, or biphasic fermentation, and the aim of these production techniques is to achieve the highest quantity of effective propagules in the shortest period of time while maintaining high quality of the desired biomass for further development of formulations (Köhl et al. 2011; Saraiva et al. 2015; Bettiol et al. 2022).

Of particular interest, submerged liquid fermentation (SmF) is more economically viable than the traditional static solid‐state or solid substrate fermentation (SSF) (Mascarin et al. 2024; Vandenberghe et al. 2021). Liquid fermentation is more advantageous for industry, as it allows greater control of the C:N ratio, pH, oxygenation (aeration), water activity, temperature, and nutrient levels, among other factors. Thus, it allows the rapid production of large quantities of submerged conidia, with a low contamination risk (Sun et al. 2014; Carvalho et al. 2018). In addition to stimulating the formation of submerged conidia, the liquid fermentation of *C. rosea* allows the production of other resistance structures, such as microsclerotia (Mascarin et al. 2022), as well as numerous bioactive secondary metabolites that play important roles in its biocontrol activity repertoire against several plant pathogenic fungi (Rodríguez et al. 2011; Saraiva et al. 2015; Sun et al. 2020; Jensen et al. 2022). The aeration rate combined with the carbon‐to‐nitrogen (C:N) ratio have been poorly investigated in submerged cultivation of *C. rosea* and could be a critical element to be managed when optimizing the production of this biocontrol agent. In addition to its versatility in providing different propagules, SmF offers opportunities to explore several secondary metabolites with antimicrobial properties to combat postharvest diseases.

*Botrytis cinerea* (Ascomycota: Sclerotiniaceae), the pathogen responsible for gray mold, affects more than 200 plant species, including tomatoes, cucumbers, eggplants, grapes, and strawberries (Elad et al. 2016). It is a major threat to the horticultural and fruit industries, causing annual economic losses estimated from 10 to 100 million USD (Brito et al. 2021). The rapid development of resistance of this fungal pathogen to multiple fungicide classes, including benzimidazoles (quinone outside inhibitors) and SDHIs (succinate dehydrogenase inhibitors), significantly hampers effective control strategies (Abbey et al. 2024; Fillinger and Walker 2016). This resistance evolution highlights the pressing need for integrated disease management strategies that reduce reliance on chemical fungicides and promote alternative control measures, including biological control options. Biological control, particularly the use of the mycoparasite *C. rosea*, offers a sustainable alternative for managing this disease. However, the antifungal activity of submerged *C. rosea* biomass and its secondary metabolites in the fermentation broth against *B. cinerea* has yet to be fully explored. In this study, we hypothesized that the aeration rate and carbon‐to‐nitrogen (C:N) ratio significantly influence the formation and production of conidia and microsclerotia in *C. rosea* during submerged cultivation. We further postulated that these propagules, together with metabolites derived from the cell‐free fermentation broth, may offer biocontrol potential against *B. cinerea*‐induced gray mold on tomatoes. This investigation enhances our understanding of how the aeration rate combined with the C:N ratio influence fungal growth and biomass production while also highlighting the role of novel bioactive propagules and secondary metabolites from fermented broth in improving the biocontrol efficacy of the mycoparasitic fungus *C. rosea* toward gray mold disease.

## 2 Materials and Methods

### 2.1 Microorganisms and Culture Maintenance

The *C. rosea* strain CMAA1284 (GenBank accession MG489966) used in these studies was isolated from rose crops in Viçosa, Minas Gerais State, Brazil, and deposited at the Embrapa Environment Collection of Microorganisms of Agricultural and Environmental Importance (CMAA). The *B. cinerea* strain Bc 39 was isolated from tomato plants and provided by Sakata Seed Sudamerica, Bragança Paulista, SP, Brazil. For the maintenance of *C. rosea*, multiplication was carried out on solid potato‐dextrose‐agar medium (PDA; Acumedia Manufacturers, Michigan, USA) supplemented with rice grains (10% w/v), and the mixture was maintained in a growth chamber at 25°C ± 1°C with a 12 h photophase for 7 days. *Botrytis cinerea* was cultured on PDA containing crushed tomato leaves in the media and maintained in a growth chamber set to 12 h photophase at 25°C ± 1°C with black light for 7 days to promote growth and sporulation.

### 2.2 Effect of Culture Aeration Rates Induced by the Medium‐to‐Volume Ratio on Submerged Cultivation of *C. rosea*

The combined effects of aeration rates and C:N ratios on morphogenesis and yield in the production of submerged conidia and microsclerotia of *C. rosea* under liquid cultivation were investigated in this assay. The aeration rate was characterized at two levels on the basis of the liquid–medium–to‐shake‐flask volume ratio (i.e., filling volume ratio), in which the high aeration rate was represented by 1/5 of the flask volume filled with liquid medium (50 mL/250 mL), and the low aeration rate corresponded to the ratio of 1:2.5 (100 mL/250 mL). In addition, two C:N ratios, 50:1 and 10:1, were tested along with aeration rates to grow *C. rosea* liquid cultures while considering the total carbon content in all the culture media fixed at 36 g of carbon/L. The assay followed a completely randomized design with nine replicates per treatment (3 independent assays × 3 replicates per treatment × 4 treatments). The liquid media were inoculated with submerged conidia produced in a liquid preculture, which, in turn, was prepared by suspending conidia scraped from fully sporulated 14‐fold colonies grown on PDA plates (Acumedia Manufacturers, Michigan, USA). The conidial suspension was prepared with 10 mL of sterilized solution of Tween 80 surfactant at 0.04% (Synth, Diadema, Brazil). The preculture received 5 mL of this fungal suspension containing 5 × 10^7^ conidia/mL in baffled Erlenmeyer flasks filled with 45 mL of potato dextrose broth (PDB, Kasvi, São José dos Pinhais, PR, Brazil), with the initial pH adjusted to 6. The liquid preculture was incubated in a rotary shaker with orbital agitation at 250 rpm (28 mm orbital diameter, Solab, Piracicaba, SP, Brazil) at 28°C ± 1°C and a 12 h photophase for 4 days.

With respect to fermentation conditions, all the liquid media tested here were inoculated with liquid precultures. Briefly, a preculture inoculum of 10 mL of submerged conidial suspension was transferred into Erlenmeyer baffled flasks filled with 90 mL of liquid medium, resulting in the treatment with 100 mL assigned to be “low aeration.” The other contrasting treatment included “high aeration” consisting of 50 mL of medium per flask, to which 5 mL of inoculum from the preculture was added to 45 mL of liquid medium. The cultures were incubated in an orbital shaker (250 rpm) set at 28°C ± 1°C with a 12 h photophase for 4 days. To determine the concentration of submerged conidia, a 1‐mL aliquot of fermented broth was taken from each culture flask and serially diluted in deionized water, and a 10–15 µL aliquot of the final dilution was transferred to a Neubauer chamber for spore counting under 400× magnification. For the quantification of microsclerotia, a 1‐mL sample of liquid culture was taken from each culture flask and diluted to 10^–1^, and then a 100‐μL aliquot of this dilution (i.e., 10^–2^) was transferred to a 75 × 25 mm glass slide covered with a 24 × 24 mm coverslip for counting only microsclerotia under the microscope at 100× magnification. The results are expressed in submerged conidia/mL and microsclerotia/mL after 2, 3, and 4 days of fermentation. The nutritional composition of the culture medium was described by Mascarin et al. (2022) (Table 1) and included dextrose monohydrate as the main carbon source, basal salts and vitamins. The nitrogen source added to the medium was whole‐grain soy flour (Nutrialy, Uberlândia, MG, Brazil), which was composed by 53% C and 6.31% N. Submerged conidia produced by the liquid preculture were always used as the inoculum source (10% v/v) in the fermentation studies with the aim at delivering a final concentration of 5 × 10^6^ conidia/mL.

**Table 1:** Composition of the liquid culture media used in the fermentation of *Clonostachys rosea* with lower and higher aeration rates at different C:N ratios.

Medium components	Preculture	T1^a^	T2	T3	T4
C:N ratio	—	10:1	50:1	10:1	50:1
Initial pH	6	6	6	6	6
Inoculum 5 × 10^7^ conidia/mL (mL)	5	5	5	10	10
Basal medium (mL)	25	25	25	50	50
Dextrose 25% (mL)	18.6	7.7	18	15.4	36
H_2_0 (mL)	1.05	12.3	2	24.6	4
Yeast extract (g)	0.35	—	—	—	—
Soybean flour (g)	—	2.12	0.42	4.24	0.84
Volume total (mL)	50	50	50	100	100

^a^ Labels for different C:N ratios and liquid medium volumes are indicated by T1, T2, T3 and T4.

### 2.3 Cell‐Free Culture Filtrate of *C. rosea*

The cell‐free culture filtrate of *C. rosea* was obtained from fermented broth produced from T2 medium (C:N ratio of 50:1 and 36 g of carbon/L). Four‐day‐old fermented broth was centrifuged for 20 min at 10,000 rpm at 10°C, after which the aqueous supernatant was filtered through a 0.22‐µm Millipore filter via a sterile syringe. This process resulted in a clear, cell‐free culture filtrate containing *C. rosea* metabolites. This crude supernatant was then preserved in a freezer at −20°C until use in antagonism bioassays with *B. cinerea*.

### 2.4 Fungal Formulation

The formulation and drying protocol employed here to obtain dry microgranules of submerged conidia and microsclerotia of *C. rosea* is described in detail by Mascarin et al. (2022). These air‐dried microgranules are easily and readily diluted in water, and the carriers and additives used in this formulation are harmless to the target pathogen or vegetable. Briefly, the fermented broth from 4‐day‐old liquid cultures was centrifuged, as previously mentioned, to remove spent medium with associated metabolites, and then, the fresh fungal biomass was used to prepare formulations for each submerged propagule, conidia or microsclerotia. To prepare these formulations, submerged conidia were harvested from culture media under a high aeration rate and a C:N ratio of 50:1, whereas microsclerotia were produced in medium with a high aeration rate and a C:N ratio of 10:1. The fungal biomass derived from each culture medium was mixed with 92.5% (w/w) diatomaceous earth (Diatom M45, Diatom Mineração Ltda., Mogi das Cruzes, SP, Brazil) and 7.5% (w/w) organosilicon‐based dispersant (Break‐thru SD260, Evonik Operations GmbH, Essen, Germany) to a final weight of 20 g of formulated product. The resulting mixture was crumbled into small pieces (< 1 mm) via a food mixer. This mixture was subsequently dehydrated via a slow drying process with controlled relative humidity (30%–50% RH) of the air flow circulanting inside an acrylic chamber for approximately 15–16 h until the final moisture content of the formulated product reached 4%–5% (w/w). After drying, these formulated fungal propagules were checked for viability and concentration by assessing colony forming units (CFUs), in which fungal colonies were counted on a selective medium composed of PDA amended with 0.001% (w/v) chloramphenicol (Sigma, Germany) and 0.01% (v/v) Triton X‐100 (Synth, Diadema, Brazil) after 2–4 days of incubation at 25°C and a 12 h photophase (Mascarin et al. 2022). The viable concentration of these formulated materials was checked before the bioassays with the pathogen and resulted in 4 × 10^8^ CFU/g for the submerged conidia‐based formulation and 4 × 10^5^ microsclerotia/g for the microsclerotia‐based formulation.

In addition, the impact of the aeration rate during the liquid growth of microsclerotia (medium T1 with “high aeration” vs. T3 with “low aeration”) on the ability of the fungus to produce aerial conidia from dry microsclerotia microgranules upon rehydration with 2% (w/v) agar–water medium was assessed. The quantification of conidia production per gram of microgranules (i.e., microsclerotial sporogenesis) was performed via the sprinkle technique (see Mascarin et al. 2022). To this end, 0.03 g of dry microsclerotia microgranules from cultures with high and low aeration rates were distributed over the entire surface of the agar–water medium (20 mL/plate) in 90 × 15 mm polystyrene Petri dishes (Pleion, Barueri, Brazil) and incubated for 7 days at 25°C ± 1°C with a 12 h photophase. The plates were covered with paraffinic plastic film (Parafilm®M) to avoid water loss during the incubation period. Immediately after sporulation, the production of conidia/g by the microsclerotia microgranules was determined with the aid of a Neubauer chamber at 400X magnification.

### 2.5 Antagonism of *C. rosea* Against *B. cinerea*: *In Vivo* Bioassay

An in vivo antagonism bioassay to assess the biocontrol efficacy of *C. rosea* on the gray mold *B. cinerea* was performed with fresh cherry tomatoes (*Solanum lycopersicum* L.) of the “Sweet Grape” variety traded by La Vita (Holambra, SP, Brazil). Tomatoes were artificially inoculated with freshly produced conidia of *B. cinerea*. For the preparation of the *B. cinerea* conidial suspension, a fully sporulated PDA culture of this fungus was scraped with 10 mL of 0.04% Tween® 80 solution, and then, serial dilution was performed for spore counting in the Neubauer chamber. Thus, 0.22 mL of this suspension with an inoculum concentration of 1 × 10^8^ conidia/mL was transferred to a flask containing 39.78 mL of 0.1% Tween 80 supplemented with 4% (w/v) sucrose, as this sugar was necessary to stimulate the germination of this fungal conidia. Before inoculation with *B. cinerea*, all tomatoes were superficially disinfected through immersion in 1% (v/v) sodium hypochlorite solution for 60 s, rinsed for 60 s in distilled water, transferred onto sterile paper towels and dried inside a laminar flow hood. After superficial disinfection, the fruits were placed in 250‐mL plastic containers covered with lids, whose bottoms were lined with sterile paper towels moistened with 1.5 mL of sterilized distilled water to maintain sufficient moisture inside the cups.

The experimental setup was designed to evaluate a preventive approach involving the use of a biocontrol agent to suppress postharvest fungal disease. The experiment followed a completely randomized design with five treatments and 22 samples (two independent trials performed with 11 biological replicates per treatment). For data analysis, the two independent trials were combined. For pathogen inoculation, two superficial cuts were made in the fruit epicarp at a depth of 5 mm via a no. 21 scalpel blade. The biocontrol agent *C. rosea* was employed as a preventive treatment on the basis of formulated propagules (submerged conidia and microsclerotia) and its cell‐free culture filtrate containing metabolites applied directly onto artificially wounded spots of tomato fruits. After 24 h of *C. rosea* application, a conidial inoculum of *B. cinerea* was applied to the same wounded spots on the tomatoes, which were then allowed to dry for several minutes inside a laminar flow hood. Briefly, these treatments included the following: T1) 15 µL of distilled water without the pathogen inoculum; T2) 15 µL of distilled water followed by a 15‐µL droplet of *B. cinerea* conidial inoculum at 5 × 10^6^ conidia/mL (positive control); T3) a *C. rosea* conidial suspension from liquid fermentation (medium T2, Table 1) at 1 × 10^8^ conidia/mL followed by the application of *B. cinerea*; T4) a *C. rosea* microsclerotia suspension at 1 × 10^4^ microsclerotia/mL from liquid fermentation (medium T1, Table 1) followed by the application of *B. cinerea*; and T5) a nondiluted cell‐free culture filtrate derived from the liquid culture T2 of *C. rosea* (high aeration and a C:N ratio of 50:1) followed by the application of *B. cinerea*. For this study, we did not test *C. rosea* treatments in the absence of *B. cinerea* because the goal here was to measure the preventive control afforded by the biocontrol agent. All treated tomato fruits were kept in an environmentally controlled chamber (BOD) set at 25°C ± 1°C, > 80% RH and a 12 h photophase. The first evaluation was performed after 5 days of incubation, and the second after 7 days, while the growth of *B. cinerea* on tomato fruits and the development of gray mold symptoms in the inoculated lesions were observed. The disease incidence was evaluated by visual diagnosis, counting the presence or absence of gray mold symptoms on the fruits, confirmed by microscopic examination of the fungal signs on the basis of morphology.

### 2.6 UPLC‐ESI‐QTOF‐MS Analysis of *C. rosea* Supernatants

The *C. rosea* fermentation broth from medium T2 (see Table 1) was centrifuged at 10,000 rpm for 20 min. The cell‐free culture filtrate (supernatant) was retrieved and then mixed with dichloromethane at a 1:2 ratio (sample:dichloromethane). The mixture was shaken at 25°C overnight. The organic fraction was then rotary evaporated to dryness (Tapfuma, Nyambo, Adu‐Amankwaah et al. 2022; Tapfuma, Nyambo, Baatjies, et al. 2022). The dried extract was resuspended in methanol and filtered through a 0.22 µm polytetrafluoroethylene (PTFE) membrane before LC injection. Chromatographic separation of the extract of *C. rosea* was performed via a Waters ACQUITY UPLC (Waters Acquity Ultra Performance LC; Milford, MA, USA) equipped with a binary solvent delivery system, an autosampler, a degasser and a thermostat column compartment. The UPLC system was coupled with a Waters Synapt Quadrupole Time–of–Flight (QQTOF) mass spectrometer (Waters Synapt HDMS; Milford, MA, USA) using a UPLC BEH C18, 1.7 μm, 2.1 × 100 mm column at 30°C. The mobile phase consisted of water with 0.1% formic acid (A) and methanol (B). The following gradient program was used: 0–5 min, 5% B; 5–10 min, 5%–40% B; 10–12 min, 40%–45% B; 12–18 min, 98% B; 18–20 min, 98% B; 20–22 min, 98% B; and 22–24 min, 5% B. The phase flow rate was 0.30 mL min^−1,^ and the injection volume was 10 μL. The ESI parameters were optimized as follows: capillary voltage, 2.0 kV; cone voltage, 30 V; ion source temperature, 110°C; desolvation gas temperature (nitrogen), 450°C; and desolvation gas flow rate (nitrogen), 500 L h^−^^1^. The instrument was operated in negative mode [M‐H]^−^^1,^ and full‐scan spectra were acquired in the *m*/*z* range of 100–1000. The SYNAPT system was calibrated with a sodium formate solution (10% formic acid:0.1% sodium hydroxide:ACN, 1:1:8, *v/v/v*), and it was used as an external reference (Lock‐Spray) for accurate mass measurements. MassLynx V4.1 software (Waters, Milford, MA, USA, 2005) was used for data acquisition and processing.

### 2.7 Data Analysis

Datasets on the production of *C. rosea* submerged propagules by different C:N ratios and aeration rates were fitted to linear mixed models (random variables assigned to flask replication from different trials) with a normal distribution, with or without interactions of fixed effects in the linear predictor. A similar statistical approach was used to assess the impact of aeration rates on the production of aerial conidia by microgranular formulations of microsclerotia upon rehydration. With respect to antagonism bioassays testing different *C. rosea* treatments on gray mold incidence on tomato fruits, the binomial data (presence vs. absence of gray mold disease) were fitted to a generalized linear mixed model (fixed effect for treatment and random effect for trial) with binomial distribution and logit link function. In all cases, when there was a significant effect of treatments or their interaction term in the model, a post hoc Tukey's HSD test was performed to test the significance of the intertreatment contrasts, and false discovery rate adjusted *P* values were used to correct for multiple testing (Benjamini and Hochberg 1995). All analyses were performed via the R statistical environment (R Core Team 2023). The generalized linear and linear mixed model analyses were performed via the package “lme4” version 1.1‐‐35.3 (Bates et al. 2015) and its extension “emmeans” version 1.10.4 (Lenth 2021).

## 3 Results

### 3.1 Influence of Aeration Rate on the Liquid Fermentation of *C. rosea*

**Figure 1:**
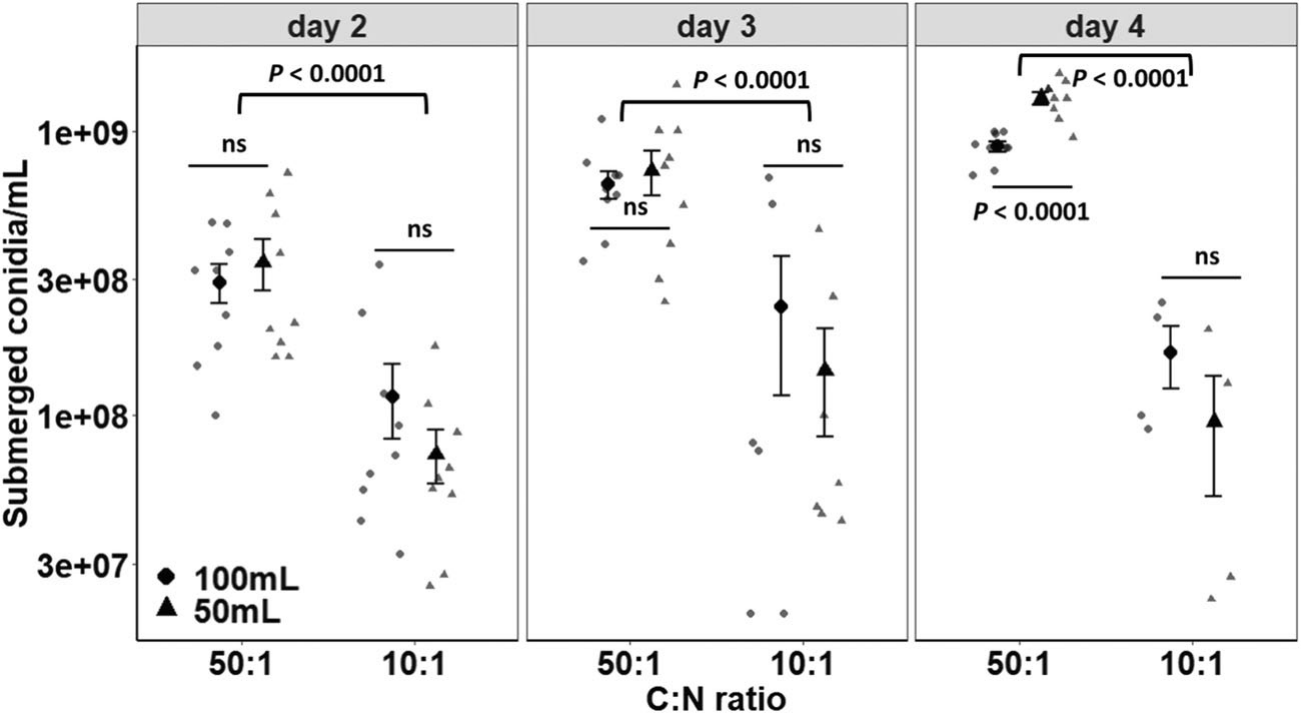
Effect of the aeration rate mediated by the working volume and the C:N ratio on the temporal production of submerged conidia of *Clonostachys rosea* under liquid cultivation in shaken flasks. The means ( ± SE, *n* = 9) were statistically significant according to Tukey's HSD test (*p* < 0.05) between aeration rates within the same C:N ratio or between C:N ratios within each aeration rate, considering the comparisons within each time interval. Otherwise, no significant (ns) differences are indicated (*p* > 0.05).

The interaction effect between cultivation time and aeration rate significantly influenced the production of submerged conidia of *C. rosea* (*F* = 3.17, *n* = 9, df = 2, 81, *p* = 0.048). Increasing aeration induced by a volume of 50 mL of liquid medium combined with a C:N ratio of 50:1 led to greater production of submerged conidia on the fourth day of fermentation, resulting in 1.31 × 10^9^ submerged conidia/mL compared with the lower aeration rate obtained with 100 mL of liquid medium (Figure 1). Another important point to maximize the production of submerged conidia was the combination of a high aeration rate and a high C:N ratio (*F* = 6.47, *n* = 9, df = 2, 81, *p* = 0.013). In this sense, highly aerated cultures of *C. rosea* increased concentrations of submerged conidia only on day 4 of cultivation. Interestingly, regardless of the aeration rate and fermentation time, the concentration of submerged conidia was always greater when the fungus was cultivated in media with a C:N ratio of 50:1, with significant increases over the cultivation time (*F* = 22.95, *n* = 9, df = 2, 81, *p* < 0.0001).

**Figure 2:**
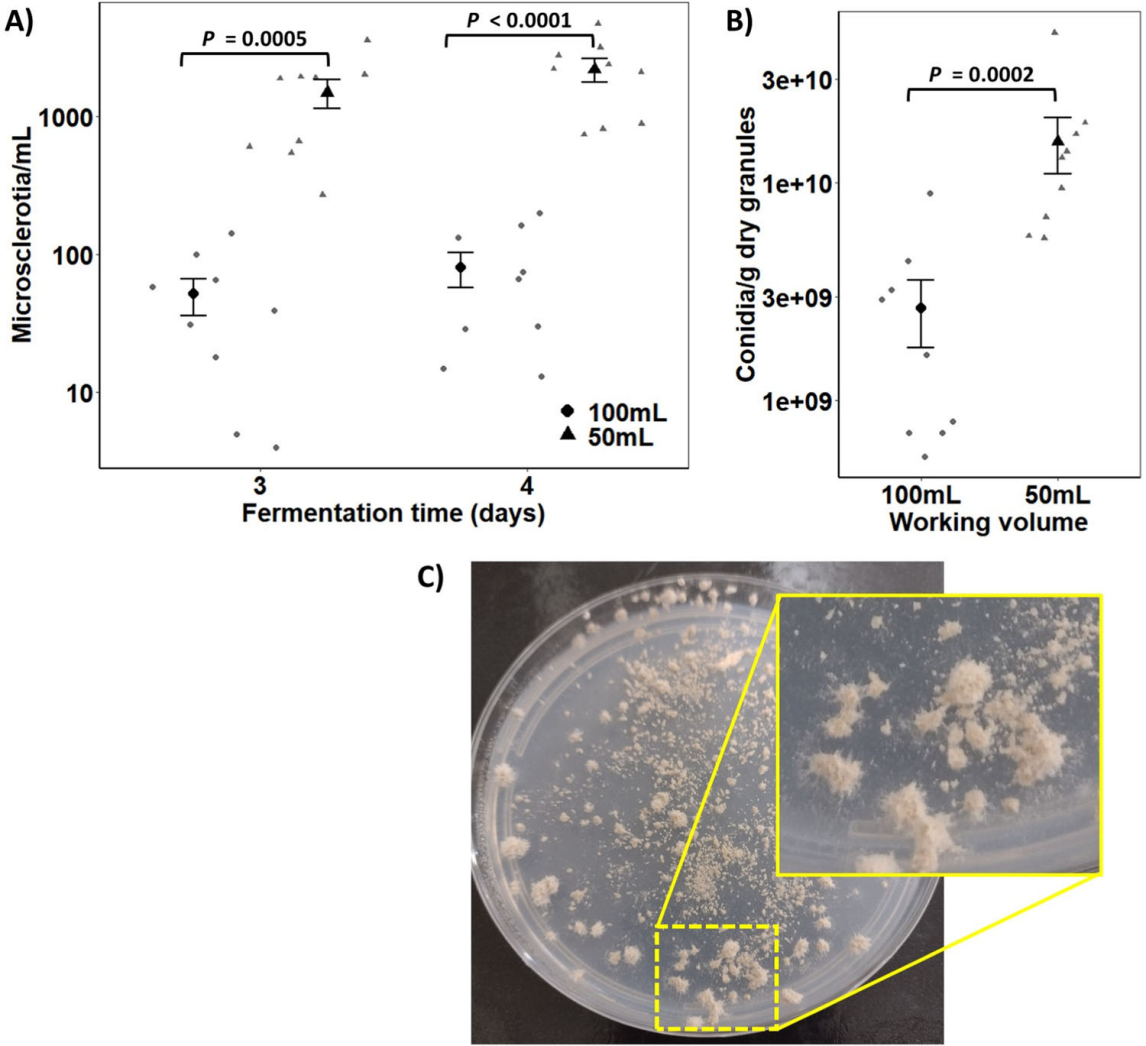
Effect of aeration rate on the production of microsclerotia (A) of *Clonostachys rosea* under submerged liquid cultivation in shaken flasks with culture media with a 10:1 C:N ratio and 36 g of C/L. The data are not shown for liquid cultures with a 50:1 C:N ratio because of the absence of microsclerotia formation. Production of aerial conidia by *C. rosea* (B) after sporulation of microsclerotial microgranules upon rehydration after 7 days on agar–water medium (C). The means ( ± SE, *n* = 9) were significantly different according to Tukey's HSD test (*p* < 0.05).

The interaction effect between aeration rate and cultivation time was not significant for microsclerotia production by *C. rosea* (*F* = 1.87, *n* = 9, df = 1, 24, *p* = 0.185), indicating that aeration rate was the only factor that influenced the microsclerotia formation rather than cultivation time. The medium with a C:N ratio of 50:1 did not induce the formation of *C. rosea* microsclerotia. However, in media with a C:N ratio of 10:1, where there is a relatively high total nitrogen content, the formation and high concentration of microsclerotia of this fungus occurred. When the volume of the medium in the flask decreased by half of the usual volume, the production of microsclerotia significantly increased (*F* = 50.41, *n* = 9, df = 1, 24, *p* < 0.0001), and there was no difference in the concentrations of this propagule between the third and fourth day of fermentation (*F* = 2.19, *n* = 9, df = 1, 24, *p* = 0.15). The highest concentrations of microsclerotia reached 1.5 × 10^3^/mL and 2.2 × 10^3^ microsclerotia/mL on days three and four of fermentation, respectively, surpassing by 29 and 27 times those concentrations obtained with a low aeration rate in 100 mL of medium (Figure 2A). Therefore, the production of microsclerotia was markedly maximized in media with a C:N ratio of 10:1 under a high aeration rate, with peak production occurring on the third day of cultivation.

The production of aerial conidia via sporogenesis (i.e., branched conidiophores formed compact, brush‐like structures) of dried microsclerotial microgranules (derived from media with a C:N ratio of 10:1) upon rehydration for 7 days significantly increased when the microsclerotia were derived from liquid cultures with high aeration rates (50 mL of media) (*F* = 23.81, *n* = 9, df = 1, 16, *p* = 0.00017; Figure 2B,C). The production of aerial conidia reached an average of 1.22 × 10^10^/g in the formulation made with 50 mL cultures compared with an average of 1.73 × 10^9^/g in the formulation derived from cultures with a lower aeration rate achieved with 100 mL of liquid medium (Figure 2B). The greater number of microsclerotia encapsulated per gram of microgranules was attributed to the higher concentration of microsclerotia attained when the fungus was cultivated at a higher aeration induced by 50 mL of liquid medium than when it was cultivated with 100 mL.

### 3.2 Biocontrol of Tomato Gray Mold

**Figure 3:**
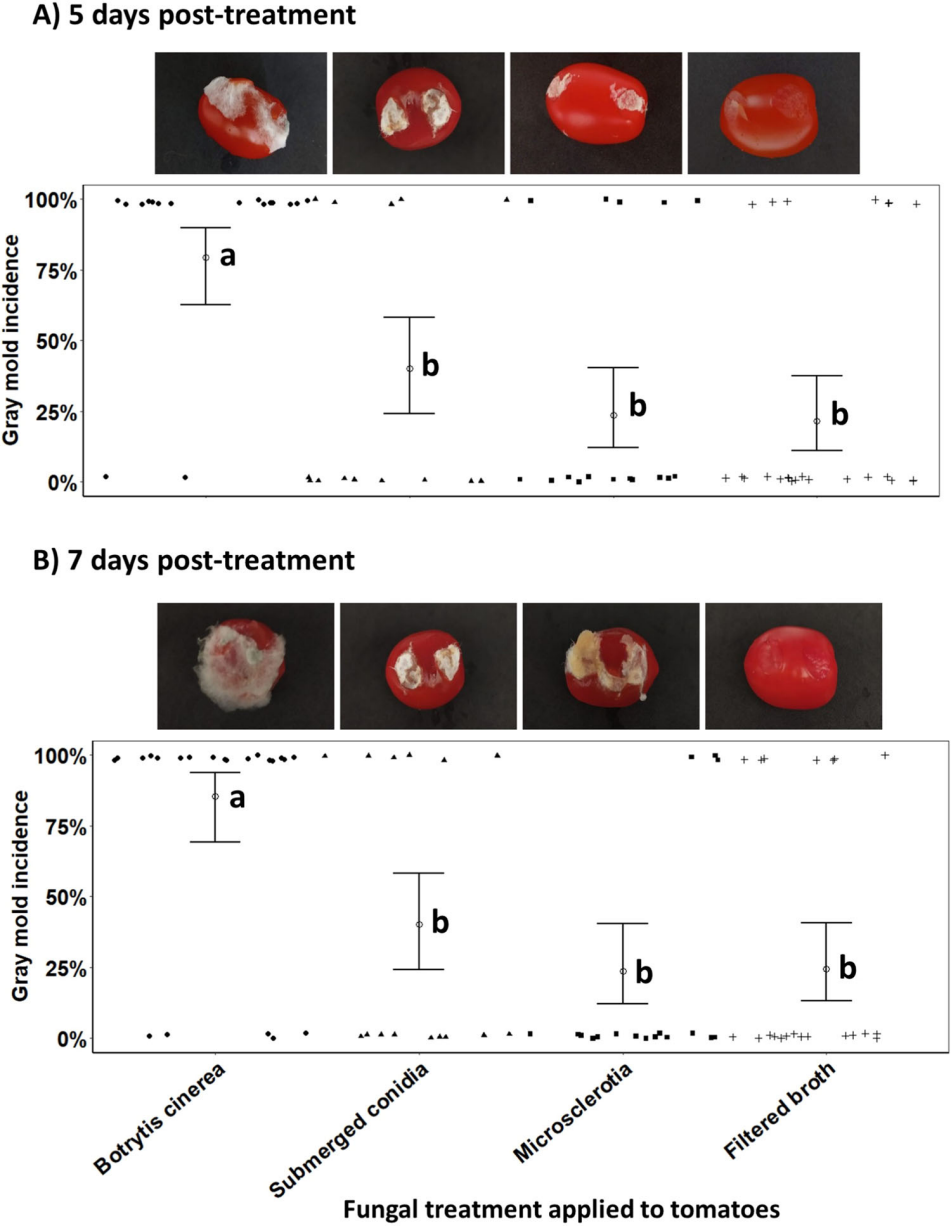
Antagonistic activity of *Clonostachys rosea* on gray mold incidence caused by *Botrytis cinerea* in tomato fruits on the fifth (A) and seventh (B) days post‐treatment. *C. rosea* treatments consisted of inoculum rates at 1 × 10^8^ submerged conidia/mL, 1 × 10^4^ microsclerotia/mL and of filtered fermented broth (crude cell‐free culture filtrate) applied directly to tomatoes immediately before the application of *B. cinerea*. Means ( ± SE, *n* = 22) followed by different letters indicate significant differences between treatments (Tukey HSD, *p* < 0.05).

On the fifth daost‐treatment, there was a significant reduction in tomato gray mold incidence due to preventive *C. rosea* treatments based on submerged propagules or cell‐free culture filtrate (*χ* ^2^ = 31.80, *n* = 22, df = 3, *p* < 0.0001). In the absence of *C. rosea* treatment, the lesions of 79% of the tomatoes inoculated with *B. cinerea* presented typical development of gray mold disease, which was notably characterized by rot. In tomato fruits that were previously treated with submerged conidia, microsclerotia or crude cell‐free culture filtrate of *C. rosea*, gray mold incidence was inhibited by 60%–78%, corresponding to disease incidences of 40%, 24% and 24%, respectively (Figure 3A).

On the seventh day post‐treatment and regardless the fungal treament applied, there was a significant protective effect of *C. rosea* on tomato fruits against *B. cinerea* infection, thus precluding gray mold development (*χ* ^2^ = 37.54, *n* = 22, df = 3, *p* < 0.0001; Figure 3B). On the other hand, tomatoes inoculated with *B. cinerea* in the absence of *C. rosea* presented 85% lesions with gray mold development and rotting aspects, whereas tomatoes previously treated with propagules or cell‐free culture filtrate of *C. rosea* presented impaired gray mold disease development, resulting in disease suppression ranging from 53% to 72%. Visually, lesions that received preventive *C. rosea* treatment with either submerged conidia or microsclerotia displaced the pathogen and were colonized by the biocontrol agent without damaging the fruits, whereas the *C. rosea* cell‐free culture filtrate had no fungal growth in the lesions and kept the lesions clean (Figure 3A,B). When tomatoes received only sterile distilled water, there was no incidence of pathogens, demonstrating that asepsis and care were needed during the experimental manipulation of the tomatoes.

### 3.3 Identification of Antifungal Metabolites in the Crude Fermentation Broth

The total ion chromatogram (TIC) of *C. rosea* organic extract obtained by liquid chromatography coupled with a QTOF mass spectrometer in negative electrospray ionization (ESI) mode identified sorbicillinoids, also called vertinoids, in *C. rosea* extract using LC‐ESI‐QTOF‐MS along with their accurate mass measurement, mass error, iFIT, molecular formula and comparison with Data ‐MS previously available in the literature.

Table 2 shows the compounds identified in the *C. rosea* extract. The accurate mass measurements of the selected peaks allowed the elemental compositions of the candidates to be calculated. To reduce the number of possible elemental compositions, we applied the iFIT approach on the basis of the isotopic pattern distribution. The screening criteria consisted of a ± 5 mDa accurate‐mass window and a lower iFIT value.

**Table 2:** Primary sorbicillinoid compounds found in the *Clonostachys rosea* organic extract derived from fermented broth produced with medium at a high aeration rate and a C:N ratio of 50:1.

m/z experimental	m/z calculated	Error (ppm)	iFit	Proposed formula	Compound
247.0964	247.697	1.7	−2.4	C_14_H_16_O_4_	Sorbicillinoide
249.1127	249.1127	0.4	0	C_14_H_18_O_4_	Hydrosorbicillinoide
263.093	263.0919	0.8	4.2	C_14_H_16_0_5_	Oxosorbicillinoide 1
511.1985	511.1968	1.6	3.3	C_28_H_32_O_9_	Oxosorbicillinoide 2
513.2143	513.2125	1.5	3.5	C_28_H_34_O_9_	Dihydrooxosorbiqinoide
527.1946	527.1917	0.6	5.5	C_28_H_32_O_10_	Bisoxosorbicillinoide

## 4 Discussion

In this study, we highlight the beneficial effect of elevated aeration rates combined with C:N ratios on the production of submerged conidia and microsclerotia from the mycoparasitic fungus *C. rosea* strain CMAA1284 via SmF. These propagules, along with cell‐free culture filtrates, exhibited significant biocontrol activity against postharvest gray mold disease caused by *B. cinerea* on tomato fruits. Our results demonstrate that the aeration rate significantly influences the morphology and yield of *C. rosea* propagules, with conidia and microsclerotia proving to be effective antifungal agents when applied as preventive treatments on tomatoes. Additionally, the cell‐free culture filtrate derived from *C. rosea* fermentation contains antifungal metabolites, primarily sorbicillinoids, which contribute to reducing gray mold incidence in tomatoes during the postharvest period. Together, the biomass and secondary metabolites present in the fermented broth offer promising bioactive components that can supplement or replace chemical fungicides for managing this postharvest disease. Our findings are consistent with previous studies showing that *C. rosea* submerged propagules effectively reduce *B. cinerea* sporulation on strawberry leaves (Carvalho et al. 2018) and parasitize *Sclerotinia sclerotiorum* sclerotia (Mascarin et al. 2022). Further studies on the short‐ and long‐term storage stability of these formulated *C. rosea* propagules are needed to assess their viability and effectiveness under various storage conditions. Moreover, fine‐tuned research exploring optimal application strategies for *C. rosea* submerged propagules and culture filtrates is necessary to effectively prevent and control gray mold on postharvest vegetables.

A key strength of this study lies in the use of a low‐cost soybean flour as the primary nitrogen source in the liquid fermentation medium for producing submerged propagules and metabolites of *C. rosea* and metabolites. With an estimated cost of only USA 1.38/kg ( ~ R$ 7.70/kg), soybean flour represents a highly economical ingredient for commercial‐scale production. In previous work, we optimized the C:N ratio using soybean flour to maximize the yield of submerged conidia and microsclerotia from the same *C. rosea* strain (Mascarin et al. 2022), achieving strong biocontrol efficacy against the whitefly *B. tabaci* and the phytopathogen *S. sclerotiorum*. Building on those findings, the present study advances this technology by demonstrating for the first time that integrating optimized aeraction with balanced C:N ratio exerts a synergistic effect on submerged conidiation or microsclerotia formation in *C. rosea* liquid cultures.

Oxygen availability during submerged cultivation of filamentous fungi is critical for dimorphic growth and biomass accumulation. In shaken flask cultures, different aeration rates can be simulated during submerged culture growth through manipulation of the agitation speed and liquid volume‐to‐flask volume ratio. Here, highly aerated liquid cultures of *C. rosea* were induced by filling 250‐mL baffled flasks with 20% liquid medium (1:5 v:v ratio), which significantly improved the yields of microsclerotia and submerged conidia. This improvement in submerged conidiation by highly aerated *C. rosea* cultures was also dictated by the C:N ratio of 50:1, with a peak in production observed by day 4 of cultivation, whereas for microsclerotia production, the proper C:N ratio was 10:1. In earlier studies, we showed that *C. rosea* required a high dissolved oxygen supply during submerged liquid growth in a benchtop bioreactor for optimal conidia production (Carvalho et al. 2018; Mascarin et al. 2022). Similar studies were performed with other important biocontrol agents, highlighting the pivotal role of high aeration rates in increasing yields of yeast‐like blastospores in the entomopathogenic fungi *Cordyceps javanica* and *Beauveria bassiana* (Jackson 2012; Mascarin et al. 2015). Furthermore, highly aerated liquid cultures had a pronounced impact on the sporogenic capacity of microsclerotial microgranules, resulting in significantly greater conidia production per gram of formulated product. This enhancement is likely attributable to either the higher concentration of microsclerotia achieved under elevated aeration or the increased accumulation of endogenous nutrient reserves within the propagules, providing additional energy to support conidiation. From a practical standpoint, this improvement may enable the use of lower product application rates of microsclerotial microgranules while still achieving effective biocontrol of soilborne and postharvest fungal pathogens.

Enhanced submerged sporulation of *C. rosea* under high aeration and elevated C:N ratio likely results from a coordinated interaction between oxidative metabolism, redox signaling, and nutrient‐sensing pathways that regulate fungal developmental transitions. Increased aeration enhances dissolved oxygen availability and respiration, leading to greater ATP turnover and moderate accumulation of reactive oxygen species (ROS). Rather than inducing oxidative damage, ROS serve as intracellular second messengers that activate MAPK cascades and transcription factors driving morphogenesis and conidiation (Hong et al. 2024). Similar oxygen‐dependent regulatory mechanisms have been documented in *Aspergillus nidulans* and *Trichoderma reesei*, where increased aeration stimulates the BrlA–AbaA–WetA network to shift growth from vegetative to reproductive stages (Cho et al. 2022). This metabolic activation also intensifies flux through the TCA cycle and oxidative phosphorylation, supporting the energetic and redox requirements for biosynthesis of conidial cell wall components and osmoprotectants.

In parallel, sporulation promotion under a high C:N ratio reflects a conserved fungal response to nitrogen limitation in carbon‐rich environments. Under these conditions, nitrogen‐sensing regulators, including AreA and the TOR pathway, repress vegetative growth while inducing developmental differentiation and secondary metabolism (Gao et al. 2007). As a result, carbon flux is redirected toward the accumulation of reserve carbohydrates (e.g., trehalose, glycogen) and precursors for chitin, β‐glucan, and lipid synthesis required for robust spore formation. Comparable trends have been reported in *Beauveria bassiana* and *Metarhizium anisopliae*, where nitrogen limitation enhances submerged conidiation and propagule vigor (Mascarin et al. 2024; Iwanicki et al. 2023).

When both aeration and C:N ratio are simultaneously increased, oxidative and nutritional signaling converge to reinforce sporogenic differentiation. High oxygen tension accelerates metabolism and amplifies ROS‐mediated developmental cues, while carbon‐excess/nitrogen‐limiting conditions impose a nutrient‐stress signal that further prioritizes reproductive development. Together, these conditions emulate the physiological transition from exponential to stationary growth phases, favoring sporulation over continued biomass expansion. Similar synergistic effects have been demonstrated in large‐scale fermentations of entomopathogenic fungi optimized for high spore yields and stability (Mascarin et al. 2024). Overall, our findings indicate that in *C. rosea*, enhanced aeration stimulates oxidative signaling and energy production, while a high C:N ratio imposes nutrient stress, thus jointly driving an efficient and coordinated submerged sporulation response.

The synergistic promotion of microsclerotia (MS) formation in *C. rosea* under high aeration and lower C:N ratios can be attributed to complementary physiological and biochemical processes similar to those reported in *Metarhizium*, *Trichoderma*, and other beneficial fungi. Increased aeration improves oxygen transfer and respiratory efficiency, supporting ATP and NAD(P)H generation and enabling the activity of oxygen‐dependent enzymes such as oxidases, peroxidases, and laccases, which are involved in cell wall cross‐linking and melanin biosynthesis (Espín‐Sánchez et al. 2023; Huarte‐Bonnet et al. 2025). These oxidative enzymes polymerize phenolic intermediates that reinforce and darken the sclerotial matrix, a hallmark of MS maturation and stress tolerance. Transcriptomic analyses in entomopathogenic fungi further confirmed the upregulation of oxidative metabolism and melanization genes during MS differentiation (Song et al. 2013; Song 2018).

In parallel, a lower C:N ratio ensures adequate nitrogen supply to support synthesis of proteinaceous and chitinous cell wall components. Nitrogen availability is essential for the production of enzymes (e.g., chitin synthases, hydrophobins, laccases) and nitrogen‐containing precursors such as N‐acetylglucosamine required for structural reinforcement and melanization (García Riaño et al. 2024; Song et al. 2013; Song 2018). Moderate nitrogen levels sustain metabolic activity while still eliciting a developmental shift toward MS formation via TOR‐mediated and nitrogen‐responsive signaling pathways. Thus, high aeration provides the metabolic energy and oxidative capacity for MS maturation, while sufficient nitrogen supplies the biosynthetic machinery and structural building blocks. Empirical optimization studies of *Metarhizium* spp. and *Trichoderma harzianum* have similarly shown that combining high dissolved oxygen with moderate nitrogen availability enhances MS yield, melanization, and desiccation tolerance (Jackson and Jaronski 2009; Kobori et al. 2015; Mascarin et al. 2014, 2022). Collectively, these findings support the conclusion that *C. rosea* responds to high aeration and lower C:N ratios with coordinated metabolic and developmental adjustments that promote efficient submerged MS production and maturation.

The fungus *C. rosea* is widely known as an efficient biocontrol agent because of its ability to suppress a wide range of plant pathogenic fungi, such as *B. cinerea* (Cota et al. 2009; Zhai et al. 2016). *Clonostachys* species are known producers of a range of secondary metabolites, mainly nitrogen‐containing compounds, terpenoids and polyketides (Han et al. 2020). A variety of polyketides occur widely in *Clonostachys*. In particular, sorbicillinoids are hexaketide metabolites with high agrochemical value due to their cytotoxic, antiviral, antibacterial, and antifungal properties (Meng et al. 2016; Hou et al. 2022). In the present study, UPLC‐ESI‐QTOF‐MS was used to elucidate the key sorbicillinoids present in *C. rosea* crude organic extracts derived from fermented broth. Some sorbicillinoids have previously been screened for antifungal activities. For example, Zhai et al. (2016) evaluated the antifungal activity of a crude extract of *C. rosea* YRS‐06 isolated from a soil sample against six target fungal species. Several sorbicillinoids (i.e., sorbicillin, trichodimerol, dihydrotrichodimerol, tetrahydrotrichodimerol, tetrahydrotrichodimer ether, and dihydrotrichodimer ether A and B) were identified in the crude extract of this fungus and displayed potent inhibitory activity against some of the target strains. Ngo et al. (2021) described the antifungal activity of EtOAc and BuOH extracts from *Trichoderma longibrachiatum* SFC100166 against several plant pathogenic fungi, including *Cladosporium cucumerinum, Colletotrichum coccodes, Cylindrocarpon destructans, Magnaporthe oyrzae*, and *Phytophthora infestans*. In the further fractionated and purified extracts, 13 compounds were identified and further classified into two groups: sorbicillinoids and terpenoids. These previous reports on the antifungal properties of sorbicillinoids align with our results concerning the remarkable bioefficacy exerted by the cell‐free culture filtrate of *C. rosea* against *B. cinerea*, thus ultimately inhibiting the incidence of tomato gray mold.

Further studies using metabolomics along with bioassay‐guided fractionation to determine the active components within the extracts are warranted to elucidate the role of these secondary metabolites from *C. rosea* crude extracts during antibiosis activity against *B. cinerea* and to understand whether these metabolites can trigger the systemic resistance response in tomatoes against *B. cinerea*. Accordingly, *C. rosea* indeed elicits resistance to gray mold in tomato fruits via a biocontrol mechanism attributed to changes in signaling molecules and protective enzymes expressed in tomatoes (Gong et al. 2016).

The growing demand for sustainable disease management underscores the importance of microbial agents that can be produced efficiently at industrial scale. Although *C. rosea* has emerged as a versatile mycoparasite and biocontrol agent, limited progress has been made toward developing cost‐effective fermentation technologies that support its commercialization. Our findings position *C. rosea* as a competitive candidate within the global biocontrol market by demonstrating that process engineering parameters, particularly aeration and C:N ratio, can be fine‐tuned to generate robust and active submerged propagules with enhanced sporogenic capacity and microsclerotia formation. This study therefore contributes to the translation of promising antagonistic fungi into reliable and scalable biocontrol formulations.

Overall, increasing the aeration rate during submerged cultivation of *C. rosea* combined with the proper C:N ratio enhances the production yields of microsclerotia and submerged conidia, providing key insights for optimizing the upscaling of this biocontrol agent. Both propagule types, along with the metabolites present in the crude cell‐free culture filtrate, strongly suppressed tomato gray mold caused by *B. cinerea*, a widely important postharvest disease of fruits and vegetables. The resulting submerged conidia, microsclerotia, and sorbicillinoids in the filtrates constitute promising biofungicidal prototypes for the sustainable management of this postharvest disease. These results reinforce the relevance of *C. rosea* within current biocontrol research and highlight its potential for integrated disease management applications. Therefore, these findings support the principles of the United Nations' One Health initiative, which contributes to more sustainable agriculture with reduced dependence on chemical pesticides and the promotion of safer and healthier food systems.

## Author Contributions

**Gabriel Moura Mascarin:** conceptualization, investigation, funding acquisition, writing – original draft, methodology, validation, visualization, writing – review and editing, formal analysis, project administration, data curation, supervision, resources. **Márcia Regina Assalin:** investigation, writing – original draft, methodology, validation, visualization, writing – review and editing, formal analysis, data curation, resources. **Nilce Naomi Kobori:** conceptualization, investigation, validation, visualization, methodology, formal analysis, writing – review and editing. **Wagner Bettiol:** writing – original draft, funding acquisition, validation, visualization, writing – review and editing, supervision.

## Ethics Statement

The authors have nothing to report.

## Consent

All the authors declare their consent to publish this article.

## Conflicts of Interest

The authors declare no conflicts of interest.

## Data Availability

The authors affirm that all the data necessary for confirming the conclusions of the article are present within the article, figures, and tables. The datasets generated during and/or analyzed during the current study are available from the corresponding author upon request.
